# Microstructure and Electrochemical Characterization of Ti-Sn Binary Alloys for Dental Applications

**DOI:** 10.3390/ma15196897

**Published:** 2022-10-05

**Authors:** Moon-Jin Hwang, Ho-Jun Song, Yeong-Joon Park

**Affiliations:** 1 GIST Central Research Facilities, Gwangju Institute of Science and Technology, Gwangju 61005, Korea; 2 Department of Dental Materials, School of Dentistry, Chonnam National University, Gwangju 61186, Korea

**Keywords:** Ti-Sn alloys, microstructure, passivation, corrosion resistance

## Abstract

This work investigated the microstructure and corrosion behaviors of Ti-Sn alloys with x wt% Sn (x = 5, 10, 15, and 20) for dental applications. The microstructures of commercially pure titanium (cp-Ti) and Ti-Sn alloys were characterized by X-ray diffractometry, optical microscopy, and transmission electron microscopy. The Vickers hardness of the Ti-Sn alloys was compared to that of cp-Ti. The corrosion behaviors of Ti-Sn alloys were tested in 0.9% NaCl solution at 37 °C using open circuit potential, potentiodynamic polarization, AC impedance, and galvanic corrosion tests. Ti-Sn alloys had a hexagonal close-packed structure and their microstructures were transformed from the equiaxed structure with irregular grain boundaries to the martensitic structure as the content of Sn in Ti-Sn alloys increased by over 15 wt%. Among the sample groups, Ti-15Sn and Ti-20Sn alloys exhibited better Vickers hardness values. Ti-Sn alloys had better corrosion resistance than cp-Ti. Ti-15Sn, which showed narrow martensitic bands, exhibited the highest corrosion resistance properties in AC impedance measurements due to its higher resistance and better capacitive parameters. Among the tested groups, the galvanic coupling of Ti-15Sn with cp-Ti showed higher corrosion potentials and lower current densities, which indicates that there was higher corrosion resistance.

## 1. Introduction

Titanium has many advantageous properties for biomaterials, such as high specific strength, corrosion resistance, and good biocompatibility. However, the problems frequently faced in clinical applications are the degradation of materials associated with fatigue, wearing, creep, and corrosion. Degradation of implant materials is one of the most important considerations in choosing materials because degraded metallic ions may provoke inflammation in the adjacent tissues [1]. Degradation could be affected by material surface topography, composition, corrosion resistance, and environmental conditions. Material surface microstructure and passive oxide film formation are closely related to corrosion resistance [2]. Passivation of the Ti surface protects the inner material by preventing the contact of the Ti substrate with biological fluids. The composition and uniformity of the passive oxide layer play an important role in the activity of Ti with biofluids. The passive oxide film formed on the Ti surface is gradually destroyed in biofluids containing chloride or fluoride ions [3,4,5]. Many efforts have aimed to enhance the mechanical or corrosion-resistant properties by developing binary Ti alloys, such as Ti-Ag, Ti-Au, Ti-Cu, Ti-In, Ti-Mn, Ti-Mo, Ti-Nb, Ti-Pd, Ti-Pt, and Ti-Zr alloys [6,7,8,9,10,11,12].

Sn as a neutral stabilizer is the nontoxic and non-allergic alloying element for use in Ti alloys [13]. 

Reports showed that Ti-Sn alloys with 1 to 30 wt% of Sn have favorable mechanical properties and can improve grindability in comparison to commercially pure titanium (cp-Ti) [14,15]. A study on the corrosion rate of Ti-Sn alloys fabricated by powder metallurgy recently reported on the evaluation of microstructure, hardness, elastic modulus, and tensile strength [16]. However, few studies have systemically investigated the corrosion properties of Ti-Sn alloys. 

This study investigated the effects of Sn addition on the microstructural properties and the electrochemical behaviors of Ti-Sn alloys and compared those properties with those of cp-Ti. The microstructural properties were investigated using X-ray diffractometry (XRD), optical microscopy (OM), transmission electron microscopy (TEM); Vickers hardness was also evaluated. The electrochemical corrosion behaviors of Ti-Sn binary alloys and cp-Ti were investigated using impedance spectroscopy, potentiodynamic polarization, and a galvanic corrosion test in 0.9% NaCl solution at 37 °C.

## 2. Materials and Methods

Titanium (99.95%, Alfa Aesar, Ward Hill, MA, USA) and Sn (99.9%) were used for fabricating Ti-Sn alloys. Cp-Ti (ASTM grade 2, Daito Steel Co. Ltd., Tokyo, Japan) was used as the control. The Ti-Sn alloys with different contents of Sn (5, 10, 15, and 20 wt%) were prepared using an arc-melting furnace (SV-230406, Seoul Vacuum Tech Co., Korea) under a vacuum condition. The final alloy ingots were obtained after repeated melting and cooling seven times to improve the homogeneity of the composition. The samples were heated with a heating rate of 5 °C/min to 150 °C lower than the solidus temperature of the respective sample groups and held for 4 hrs for homogenization treatment using a vertical tube furnace (HK-9906, Kibaek Industrial Furnace Co., Ltd., Hwaseong, Korea) in an argon environment. The samples were furnace-cooled to 600 °C, followed by air-cooling to room temperature. The samples were embedded in epoxy resin and cut to 1.2 mm thickness using diamond grinding disks.

The crystal structures were measured using a high resolution X-ray diffractometer (HR-XRD; X’Pert Pro, PANalytical, Almelo, The Netherlands) with a Cu 2KW target (Max. 60 kV, 55 mA) at a scan rate of 0.02°/s in the range of 20~90° 2θ. The mosaic-stitching tiled images were obtained using an optical microscope at ×50 magnification with iSolution Lite software (IMT i-solution Inc., Vancouver, BC, Canada). The stitching images were created automatically by aligning several microscopic images into a single mosaic image. The differences in brightness between images were eliminated using the auto image-stitching function.

The microstructures were observed using HR-TEM (Technai-F20, FEI, Eindhoven, The Netherlands).

The Vickers hardness test was performed on the samples using a Vickers microhardness tester (Postfach4350, Zwick, Ulm, Germany) with 500 g-loading for 30 s.

For corrosion tests, 10 mm-diameter samples were obtained using wire cutting and polishing by 2000-grit SiC paper. The electrochemical corrosion behaviors of Ti-Sn alloys and cp-Ti were investigated using potentiodynamic polarization, impedance spectroscopy, and a galvanic corrosion test in 0.9% NaCl solution at 37 ± 1 °C. 

The impedance of the samples was measured using an impedance spectrometer (ZIVE sp2, WonA Tech Co., Ltd., Seoul, Korea) following the ASTM G106-89 method [17]. The 0.2826 cm^2^ of the sample surface was exposed to the electrolyte through the opening on a tightly sealed Teflon mold assembly. From the exposure of the sample surface to the electrolyte, the open circuit potential was measured for 1 h. The impedance spectra were obtained in the frequency range of 10^6^–10^−2^ Hz with 10 mV amplitude. The parameters of the equivalent circuit, obtained using non-linear least-square fitting, were calculated using impedance analysis software (Zman 2.2, WonA Tech Co., Ltd., Seoul, Korea).

Potentiodynamic polarization measurements were performed with a three-electrode technique using the test samples as the working electrode, the high-density carbon counter electrode, and the saturated calomel reference electrode [18]. Before the tests, the electrolyte was purged with Ar gas for 20 min at a flow rate of 150 mL/min to remove dissolved oxygen. The potentiodynamic polarization curves were obtained using Potentiostat (WAT100, WonA Tech Co., Ltd., Seoul, Korea) in the range from −1.5 to 1.5 V (vs. SCE) at a scan rate of 5 mV/s. Triplicate samples were tested. For each measurement, a fresh electrolyte was used. 

Galvanic corrosion between cp-Ti and Ti-Sn alloys was evaluated. The epoxy resin-embedded 10 mm-diameter sample surfaces were polished with 2000 grit SiC paper, which was followed by cleansing with distilled water in an ultrasonic cleaner for 5 min. Just before each galvanic corrosion measurement, the sample surfaces were polished with 4000 grit SiC paper. The distance between galvanic couples was maintained at 1 cm. 

## 3. Results and Discussion

**Figure 1:**
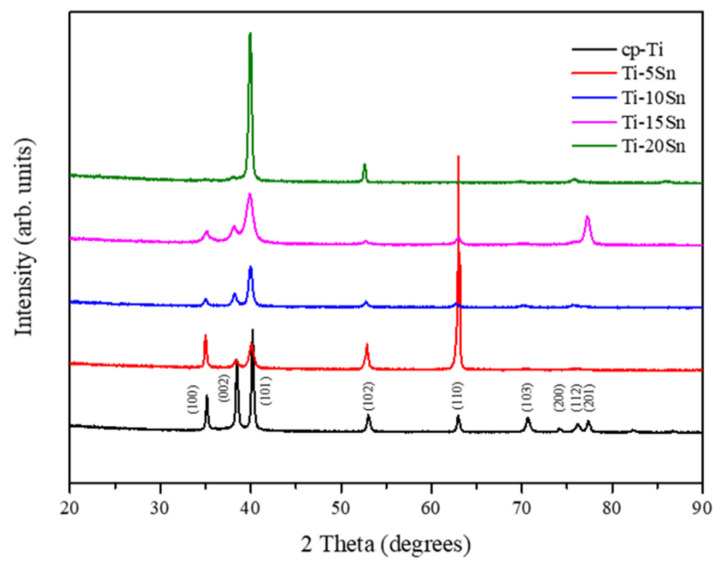
XRD patterns of cp-Ti and Ti-xSn alloys (x = 5~20 wt%).

The Ti-Sn alloys exhibited a hexagonal closely packed (hcp) α phase structure with no other phase presence, as did cp-Ti (Figure 1). The differences in the diffraction intensity and the peak width of the Ti-Sn alloys are possibly related to the difference in texture deformation with Sn content changes.

**Figure 2:**
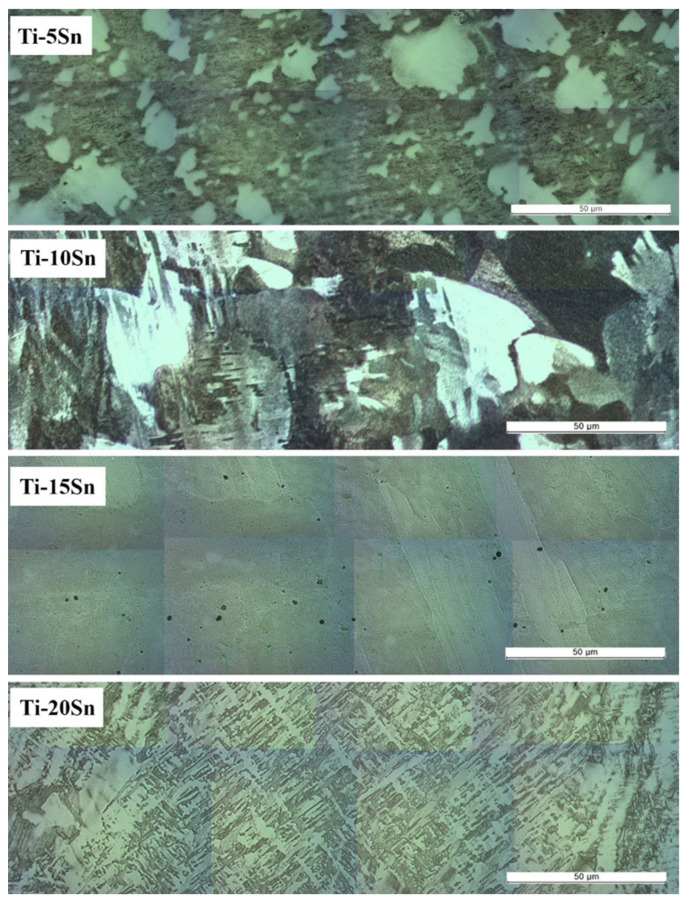
The mosaic-stitching optical microscope images of Ti-xSn alloys (x = 5~20 wt%). Original magnification was ×50.

**Figure 3:**
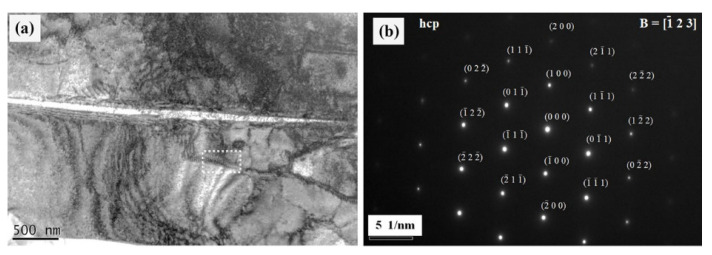
(**a**) TEM image of Ti-20Sn alloy and (**b**) SAED pattern for the selected region (white dotted rectangle).

Figure 2 shows the mosaic-stitched tiled optical microscope images of Ti-Sn alloys after etching with Kroll’s reagent. The microstructure of the Ti-Sn alloys was changed from an equiaxed structure to a martensitic structure with the increase of Sn content. It was observed that the sizes of irregular grains of Ti-5Sn increased in Ti-10Sn. From Ti-15Sn, the martensitic structure began to appear in the matrix, and Ti-20Sn had a predominantly martensitic structure. As shown in Figure 3, the acicular martensite phase with an hcp structure was confirmed by the SAED pattern in Ti-20Sn.

As listed in Table 1, Ti-Sn alloys showed higher Vickers hardness values (335~450 kgf/mm^2^) than cp-Ti (154 kgf/mm^2^). A similar increasing trend was observed for Ti-Sn alloys with 5~10 wt% and 15~20 wt% of Sn content. Ti-15Sn and Ti-20Sn alloys exhibited significantly higher Vickers hardness values (442~450 kgf/mm^2^) than cp-Ti, possibly attributable to their martensitic structure development [19,20,21,22].

**Figure 4:**
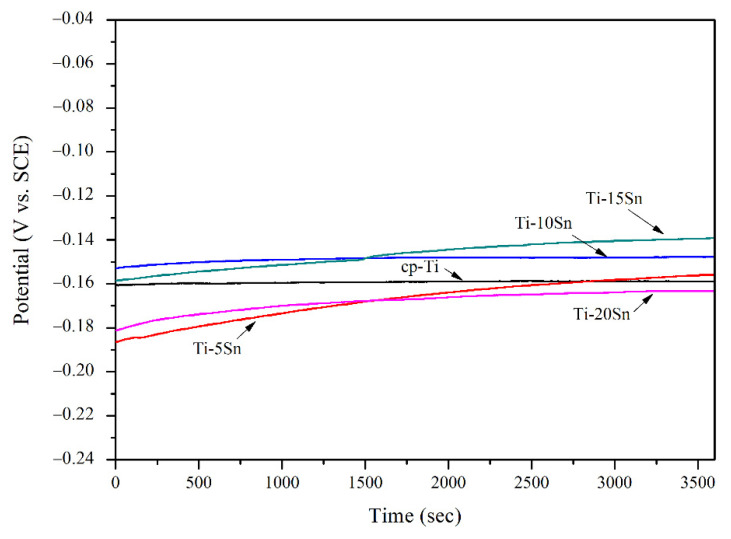
The open circuit potential curves of cp-Ti and Ti-Sn alloys in 0.9% NaCl solution at 37 °C.

The open circuit potential (OCPs) vs. time curves of Ti-Sn binary alloys and cp-Ti are shown in Figure 4. The OCP of cp-Ti was maintained at about −0.16 V during the measurement period, while the OCPs of Ti-Sn samples increased gradually with time. The difference between the initial potential and the final potential at 3600 s indicates the ability of the Ti-Sn alloys to form a passive oxide layer on the alloy surfaces. The OCP curves of Ti-Sn alloys clearly demonstrate their higher passivation capacity compared to cp-Ti. The initial OCPs of Ti-5Sn and Ti-20Sn started at −0.186 V and −0.181 V, respectively, which were lower than those of cp-Ti. However, as the measurement time increased, the OCP of Ti-5Sn increased to a slightly higher value than that of cp-Ti after 3000 s, while Ti-20Sn had the lowest potential among tested groups after 1600 s. Ti-10Sn and Ti-15Sn displayed nobler OCPs than cp-Ti throughout the measurement period. The electronegativity of Ti (1.54) is lower than that of Sn (1.94). It is expected that Sn atoms in the Ti-Sn alloy attract the electrons of Ti atoms and the potentials of Ti-Sn alloys increase with the increase of Sn content. The final OCPs of Ti-Sn alloys increased sequentially from Ti-5Sn to Ti-15Sn. However, Ti-20Sn showed the lowest OCP value, which is attributed to enhanced cathodic reaction of Ti-20Sn, as seen in Figure 7 [23].

**Figure 5:**
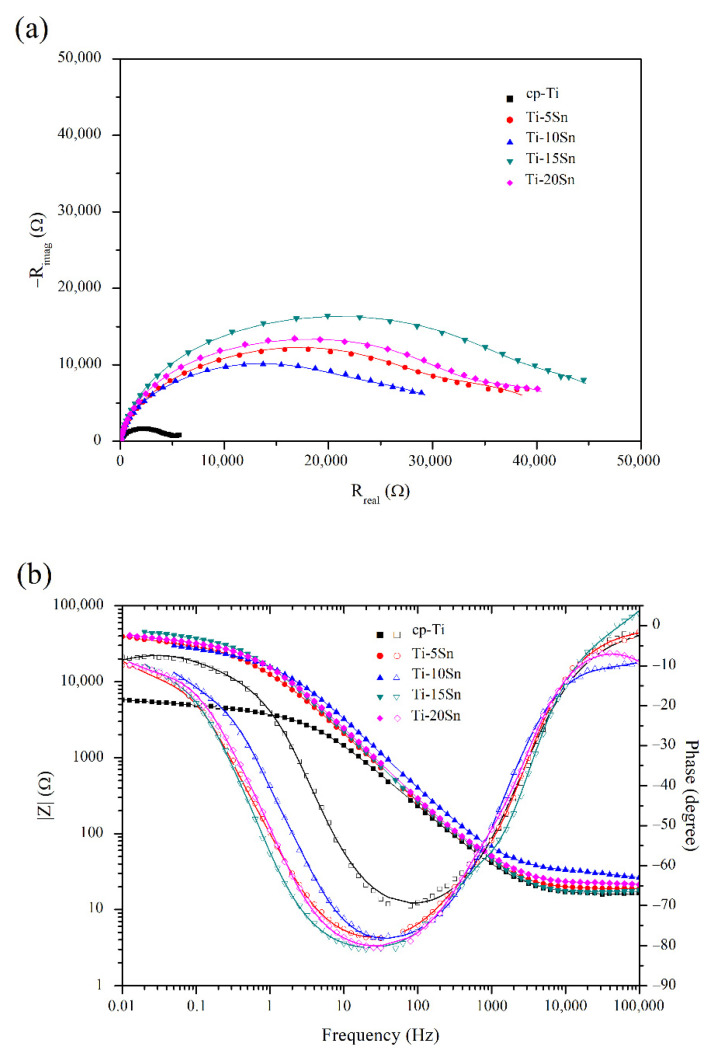
The (**a**) Nyquist and (**b**) Bode impedance curves of cp-Ti and Ti-Sn alloys in the range from 10^6^ Hz to 10^−2^ Hz in 0.9% NaCl solution at 37 °C.

Figure 5 shows the Nyquist (a) and Bode (b) impedance spectra of the Ti-Sn alloys and cp-Ti. Ti-Sn alloys exhibited superior impedance spectra compared to cp-Ti. In Figure 5a, all samples showed similarly depressed semi-circles, which indicated that a porous layer formed on the samples. As the Sn content in Ti-Sn alloys changed from 5 to 20 wt%, the diameter of the semicircles showed fluctuating results, with the highest value in the 15 wt% group. The phenomenon that Ti-15Sn displayed a semicircle with a wider diameter implies its higher charge transfer resistance. 

Figure 5b demonstrates the relationship between frequency vs. impedance magnitude and phase difference. The curves in the high-frequency range correspond to the resistance of the solution, the middle-frequency range corresponds to the properties of passive layers, and the low-frequency range corresponds to the interface properties between the metal surface and the passive layer [24]. On the frequency vs. phase curves of cp-Ti and Ti-Sn alloys, the samples had more than one constant phase element (CPE). The slope of the curves of frequency vs. impedance magnitude implies that the passive layers exhibit a capacitive property. The passive layer with ideal capacitance shows a slope of −1 and a phase difference of −90° [25]. As shown in Figure 5b, Ti-Sn alloys showed enhanced properties of passive layers compared to cp-Ti. Ti-Sn alloys had slopes of −0.80~−0.85 and phase differences of −71 ~ −76°, while cp-Ti had a slope of -0.76 and a phase difference of −69°. Ti-15Sn showed better passive layer properties in the low-frequency range with a lower phase difference and higher impedance magnitude. In contrast, Ti-10Sn displayed the worst properties of passive layers among Ti-Sn alloy groups with a phase difference of −71°. 

**Figure 6:**
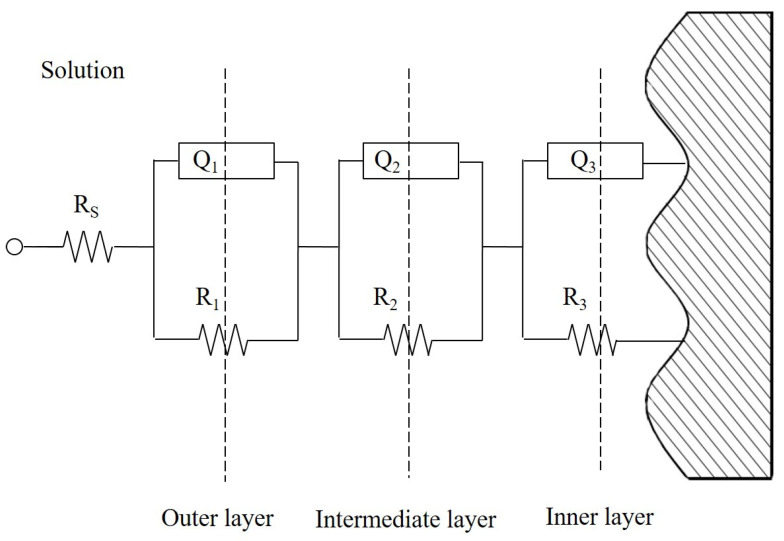
Equivalent circuit for fitting the impedance spectra of cp-Ti and Ti-Sn alloys. R_s_, R_1_, R_2_, and R_3_ are the resistance of solution, outer layer, intermediate layer, and inner layer, respectively. Q_1_, Q_2_, and Q_3_ are the CPEs of the outer, intermediate, and inner layers, respectively.

The equivalent circuit model best fitting to the impedance spectra of the Ti-Sn alloy of this work is shown in Figure 6. The best fit model showed the formation of a stratified protective passive layer on the surface of the sample [26]. The outer layer was formed by corrosion products of an intermediate layer. The electrochemical parameters are shown in Table 2. The impedance of CPE (Z_CPE_) is defined as follows:

$$Z_{\mathrm{CPE}}=\frac{1}{Q{\left(j\omega \right)}^{n}}$$

where, ω, j, and n, respectively, denote the angular frequency, imaginary number, and slope (|Z| vs. frequency) of the impedance Bode plot. In general, the capacitance is inversely proportional to the thickness of the passive layer. The lower n and higher Q values indicate poor capacitive behavior due to increased roughness, pores, or cracks in the passive layer [27]. The parameters are determined by the porosity or microstructure of the layers of samples. 

**Figure 7:**
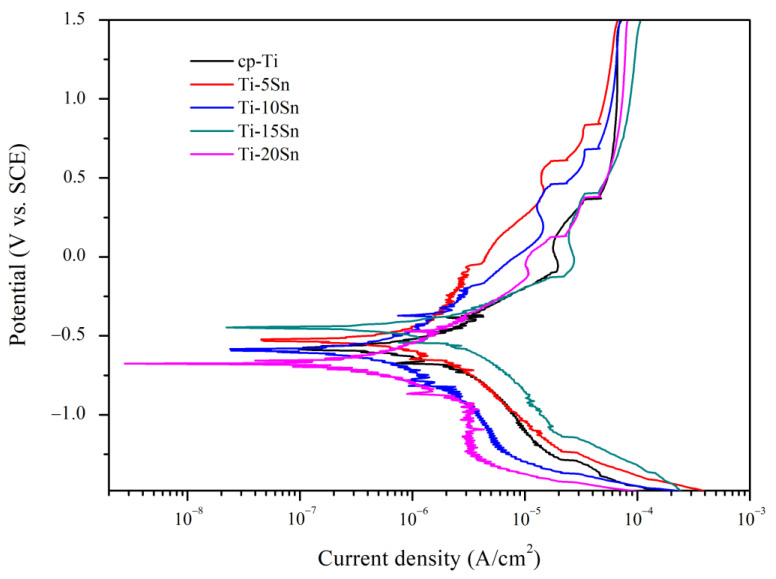
The potentiodynamic polarization curves of cp-Ti and Ti-Sn alloys in the range of −1.5 V to 1.5 V vs. SCE in 0.9% NaCl solution at 37 °C.

Based on the impedance parameters shown in Table 2, cp-Ti had comparatively lower resistance in all layers and showed porous passive layer behavior in the outer and intermediate layer. Ti-15Sn alloys had relative high resistance and capacitive parameters in the outer and intermediate layers but showed relatively small thickness and porous property in the inner layer. In the intermediate layer, Ti-Sn alloys showed a gradual increase in capacitive parameters in the CPE2 and n2 values as the Sn content increased, except for the Ti-10Sn alloy. Ti-20Sn had a relatively good resistance and capacitive properties in the outer and intermediate layer but showed relatively weak inner layer properties with the lowest value of *n* = 0.72, which could be attributed to the current oscillation behavior and passive layer breakdown that was observed in the potentiodynamic anodic polarization curve of Ti-20Sn (Figure 7). 

Figure 7 shows the representative potentiodynamic polarization curves of cp-Ti and Ti-Sn binary alloys. As the potential increased in the anodic direction, the potentiodynamic anodic polarization was characterized by the following steps: oxidation of electrolyte, primary passivation, repassivation, and trans-passivation. For cp-Ti, the oxidation reaction of the electrolyte occurred on the surface from zero-current potential (−0.58 V) to active–passive transition potential (−0.10 V). The primary passivation reaction started from −0.10 V, and the current density was 1.78 × 10^−5^ A/cm^2^ at 0.06 V. With further increase in the potential range above 0.37 V, a rapid increase of current density was observed due to the breaking of the passive layer and localized corrosion. After the breakdown and corrosion of the passive layer, the repassivation reaction occurred above 0.37 V. The cp-Ti exhibited stable repassivation behavior at a wide range and the current density was 6.61 × 10^−5^ A/cm^2^ at 1.25 V. 

Ti-Sn alloys showed a similar potentiodynamic anodic polarization behavior to cp-Ti, but their repassivation behaviors differed from cp-Ti. After the breakdown of the primary passive layer of the Ti-Sn alloys, they showed repassivation behaviors in a narrow range due to the breakdown of the re-passive layer and localized corrosion followed by trans-passive behavior. This finding indicated that the passive layer of Ti-Sn alloys consisted of more than one layer. The primary passivation position of Ti-5Sn and Ti-10Sn shifted to the positive direction compared to cp-Ti. Table 3 lists the potentiodynamic polarization parameters obtained by the Tafel extrapolation method. Polarization resistance was calculated from the parameters obtained from the Tafel plot using the following Equation (1) [28]:

I_corr_ = β_a_ × β_c_/2.3R_p_(β_a_ + β_c_) (1)

where Icorr, βa, βc, and Rp mean corrosion current density, anodic Tafel slope, cathodic Tafel slope, and polarization resistance, respectively.

The corrosion rate (C_R_, metal dissolution rate) was calculated using the following Equation (2):

C_R_ = (I_corr_ × K × E_w_)/(A × d) (2)

where K, Ew, A, and d mean corrosion rate constant (3272 mm/year), equivalent weight, area, and density, respectively. Ti-Sn alloys had similar E_corr_ (−519~−577 V) up to 15 wt% of Sn content, mostly higher than that (−574 V) of cp-Ti, while Ecorr of Ti-20Sn decreased significantly to −723 V. 

Alloying Sn with Ti had positive effects on corrosion resistance in view of I_corr_, R_p_, and corrosion rate. Ti-10Sn and Ti-20 exhibited lower I_corr_ than those of Ti-5Sn and Ti-15Sn. From the calculations of Equations (1) and (2), Ti-10Sn and Ti-20 had higher polarization resistance and lower corrosion rates than Ti-5Sn and Ti-15Sn.

**Figure 8:**
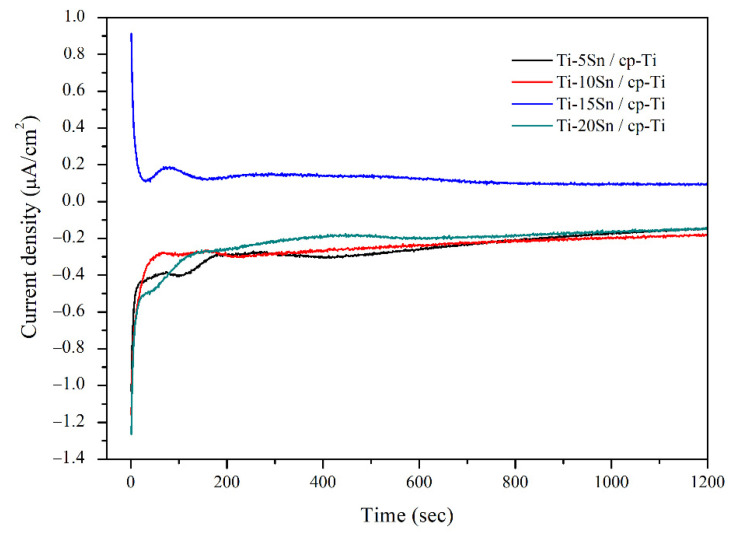
The galvanic current curves of Ti-Sn alloy/cp-Ti couples in 0.9% NaCl solution at 37 °C.

Figure 8 represents the galvanic corrosion behaviors of Ti-Sn alloys/cp-Ti during the test periods. Ti-15Sn/cp-Ti exhibited notably positive current densities throughout the test period, which indicated better corrosion resistance, while Ti-5Sn, Ti-10Sn, and Ti-20Sn coupled to cp-Ti had negative current densities. The positive galvanic current value indicates less corrosion propensity attributed to the cathodic reaction on Ti-15Sn coupled to cp-Ti [29]. It is attributed to the notable difference in potentials between Ti-15Sn and cp-Ti, as shown in Figure 4 and Figure 7. The other Ti-Sn alloys showed negative galvanic current values, which indicated the anodic reaction on Ti-Sn alloys coupled to cp-Ti. In the galvanic current density vs. time curves, fluctuating results were observed from initial time to 600 s; subsequently, current densities were maintained at relatively constant levels. Table 4 shows the galvanic current density of Ti-Sn alloy/cp-Ti combinations. Ti-15Sn showed a stable current density (0.08 × 10^−6^ A/cm^2^) after 600 s. Ti-5Sn/cp-Ti and Ti-20Sn/cp-Ti had the same current density (−0.14 × 10^−6^ A/cm^2^) at 1200 s. Ti-10Sn/cp-Ti had a relatively lower current density (−0.17 × 10^−6^ A/cm^2^) than other combination groups at 1200 s.

## 4. Conclusions

The study investigated the microstructure and electrochemical corrosion behaviors of Ti-Sn alloys with 5~20 wt% Sn for dental applications. The passivation layer forming ability was influenced by the microstructure of Ti-Sn alloys. With increased Sn content, the equiaxed structures of Ti-5Sn and Ti-10Sn changed to martensitic structures in Ti-15Sn and Ti-20Sn alloys. For Ti-Sn alloys, adding Sn to Ti improved the hardness and increased the corrosion resistance compared to cp-Ti. Ti-Sn alloys with 5, 10, and 15 wt% Sn content exhibited higher open circuit potentials. From the analysis of Tafel plots, Ti-Sn alloys with 5, 10, and 15 wt% Sn had higher corrosion potentials and lower corrosion densities compared to the −574 mV and 0.611 × 10^−6^ A/cm^2^ of cp-Ti. Although the corrosion potential of Ti-20Sn was as low as approximately −700 mV, it had a lower corrosion density. Results of the impedance analysis showed the highest corrosion resistance in Ti-15Sn due to high resistance and capacitive parameters in the outer and intermediate layers of Ti-15Sn. The galvanic coupling between Ti-15Sn and cp-Ti displayed positive current densities throughout the test period, while Ti-5Sn, Ti-10Sn, and Ti-20Sn coupled with cp-Ti had negative current densities. The study concluded that Ti-15Sn had promising passivation properties.

## Figures and Tables

**Table 1:** Vickers hardness values of cp-Ti and Ti-xSn alloys (x = 5~20 wt%) (*n* = 5).

Sample	Vickers Hardness (kgf/mm^2^)
cp-Ti	154.44 ± 5.93
Ti-5Sn	342.20 ± 61.28
Ti-10Sn	334.80 ± 29.08
Ti-15Sn	449.60 ± 73.59
Ti-20Sn	442.00 ± 22.45

**Table 2:** The impedance parameters of cp-Ti and Ti-Sn alloys (*n* = 3).

Sample	Solution	Outer Layer			Intermediate Layer			Inner Layer		
	Rs (Ω)	R1 (kΩ)	CPE1 (µ S⋅s^n1^)	n1	R2 (kΩ)	CPE2 (µ S⋅s^n2^)	n2	R3 (kΩ)	CPE3 (µ S⋅s^n3^)	n3
cp-Ti	15.3 (1.5)	2.53 (1.48)	2367 (3611)	0.76 (0.06)	7.62 (6.14)	4216 (3973)	0.78 (0.07)	2.55 (1.19)	183 (271)	0.80 (0.05)
Ti-5Sn	14.6 (4.8)	11.05 (8.96)	509 (756)	0.88 (0.42)	5.75 (2.39)	977 (1476)	0.87 (0.09)	8.89 (6.43)	117 (177)	0.85 (0.07)
Ti-10Sn	21.1 (2.4)	7.13 (9.07)	269 (295)	0.70 (0.03)	9.92 (5.00)	1580 (1360)	0.76 (0.18)	2.87 (1.87)	15 (4)	0.93 (0.06)
Ti-15Sn	14.8 (1.9)	16.30 (12.60)	570 (860)	0.84 (0.10)	9.41 (8.70)	21 (12)	0.91 (0.03)	10.98 (5.92)	1030 (1070)	0.80 (0.12)
Ti-20Sn	19.9 (4.2)	9.71 (7.39)	81 (62)	0.79 (0.13)	6.28 (6.30)	14 (2)	0.95 (0.04)	15.50 (3.77)	710 (271)	0.72 (0.08)

S and s are abbreviations for Siemens and second, respectively. Rs, R, CPE, and n represent solution resistance, resistance of passive layers, constant phase element, and bode plot slope (|Z| vs. frequency), respectively. Standard deviations are in the parentheses.

**Table 3:** Potentiodynamic polarization curve analysis results using the Tafel extrapolation method (*n* = 3).

Sample	E_corr_ (mV)	I_corr_ (×10^−6^ A/cm^2^)	β_a_	β_b_	R_p_ (×kΩ)	Corrosion Rate (×10^−2^ mm/year)
cp-Ti	−574 ± 41	0.611 ± 0.171	0.267 ± 0.091	0.188 ± 0.179	78.7 ± 10.5	1.88 ± 0.53
Ti-5Sn	−530 ± 43	0.407 ± 0.066	0.199 ± 0.013	0.163 ± 0.021	97.8 ± 21.6	1.25 ± 0.20
Ti-10Sn	−577 ± 110	0.254 ± 0.171	0.187 ± 0.035	0.146 ± 0.027	193.9 ± 124	0.78 ± 0.53
Ti-15Sn	−519 ± 55	0.325 ± 0.149	0.186 ± 0.013	0.143 ± 0.021	123.8 ± 54	1.00 ± 0.46
Ti-20Sn	−723 ± 102	0.196 ± 0.103	0.173 ± 0.06	0.159 ± 0.021	223.0 ± 119	0.61 ± 0.32

**Table 4:** Galvanic current densities of Ti-Sn alloy/cp-Ti couples versus test time (*n* = 3).

Sample	Current Density (μA/cm^2^)						
	1 s	5 s	10 s	60 s	600 s	1200 s	Integrate
Ti-5Sn	−1.65 ± 0.65	−1.04 ± 0.36	−0.78 ± 0.24	−0.41 ± 0.08	−0.23 ± 0.06	−0.14 ± 0.03	297.72 ± 57.35
Ti-10Sn	−1.34 ± 0.29	−0.86 ± 0.21	−0.69 ± 0.18	−0.40 ± 0.10	−0.22 ± 0.09	−0.17 ± 0.10	298.33 ± 106.82
Ti-15Sn	1.10 ± 0.21	0.611 ± 0.17	0.40 ± 0.15	0.13 ± 0.07	0.08 ± 0.04	0.07 ± 0.03	121.33 ± 64.36
Ti-20Sn	−1.67 ± 0.46	−1.09 ± 0.27	−0.85 ± 0.18	−0.46 ± 0.08	−0.20 ± 0.08	−0.14 ± 0.06	282.38 ± 91.56

## Data Availability

The data presented in this study are available on request from the corresponding author.
